# Scoring System to Evaluate the Performance of ICU Ventilators in the Pandemic of COVID-19: A Lung Model Study

**DOI:** 10.3389/fmed.2021.663608

**Published:** 2021-07-14

**Authors:** Xingshuo Hu, Fei Xie, Kaifei Wang, Hongjun Gu, Guoxin Mo, Ruoxuan Wen, Ying Zhao, Qingyun Yang, Knut Möller, Zhanqi Zhao, Lixin Xie

**Affiliations:** ^1^Critical Care, College of Pulmonary and Critical Care Medicine, Chinese PLA General Hospital, Beijing, China; ^2^Institute of Technical Medicine, Furtwangen University, Villingen-Schwenningen, Germany; ^3^Department of Biomedical Engineering, Fourth Military Medical University, Xi'an, China

**Keywords:** scoring system, ventilator performance, stability of pressurization, leakage correction, accuracy in volume delivery, performance of triggering, analytic hierarchy process

## Abstract

Ventilators in the intensive care units (ICU) are life-support devices that help physicians to gain additional time to cure the patients. The aim of the study was to establish a scoring system to evaluate the ventilator performance in the context of COVID-19. The scoring system was established by weighting the ventilator performance on five different aspects: the stability of pressurization, response to leaks alteration, performance of reaction, volume delivery, and accuracy in oxygen delivery. The weighting factors were determined with analytic hierarchy process (AHP). Survey was sent out to 66 clinical and mechanical experts. The scoring system was built based on 54 valid replies. A total of 12 commercially available ICU ventilators providing non-invasive ventilation were evaluated using the novel scoring system. A total of eight ICU ventilators with non-invasive ventilation mode and four dedicated non-invasive ventilators were tested according to the scoring system. Four COVID-19 phenotypes were simulated using the ASL5000 lung simulator, namely (1) increased airway resistance (IR) (10 cm H_2_O/L/s), (2) low compliance (LC) (compliance of 20 ml/cmH_2_O), (3) low compliance plus increased respiratory effort (LCIE) (respiratory rate of 40 and inspiratory effort of 10 cmH_2_O), (4) high compliance (HC) (compliance of 50 ml/cmH_2_O). All of the ventilators were set to three combinations of pressure support and positive end-expiratory pressure levels. The data were collected at baseline and at three customized leak levels. Significant inaccuracies and variations in performance between different non-invasive ventilators were observed, especially in the aspect of leaks alteration, oxygen and volume delivery. Some ventilators have stable performance in different simulated phenotypes whereas the others have over 10% scoring differences. It is feasible to use the proposed scoring system to evaluate the ventilator performance. In the COVID-19 pandemic, clinicians should be aware of possible strengths and weaknesses of ventilators.

## Introduction

Ventilator in the intensive care units (ICU) is life-support device that helps physicians gaining additional time to cure the patients. Several previous studies have compared the performance of different ventilators regarding triggering (1), system leaks (2, 3), and accuracy in volume and pressure delivery (4). The performance varied from device to device, and depended on the testing items. One device might be accurate in volume but not in pressure delivery. A complex scoring system to evaluate the overall performance of ventilator regarding various aspects is missing.

The outbreak of the novel coronavirus disease 2019 (COVID-19) has spread rapidly around the world (5). About 19% of patients in China developed hypoxic respiratory failure and required certain level of ventilation support (6).The situation in other countries is similar. Patients with COVID-19 show various phenotypes that may require different respiratory treatments, characterized as low compliance (LC) due to lung collapse or high airway resistance due to inflammation and mucus (7, 8). The performance of ventilator could be various for different phenotypes, which was not well-studied. Since the number of infected patients is large and still increases dramatically, a large number of ventilators required to support patients' respiratory system (9). New companies are recruited to build ventilators, which might have no experience on manufacturing ventilators or even medical devices prior to the pandemic. A well-designed scoring system may be helpful for the evaluation and improvement of the ventilators.

The aim of the study was to establish a scoring system to evaluate the ventilator overall performance, as well as different aspects. Based on the proposed scoring system, ICU most commonly used ventilators in China were compared in order to demonstrate the feasibility of the novel scoring system. We hypothesized that the performances of the ventilators evident.

## Materials and Methods

The scoring system was established using the analytic hierarchy process (AHP) (10). The AHP hierarchy consisted of five criteria regarding the ventilator performance, which were selected based on our experiences and previous studies (2–4).

### The Stability of Pressurization

The stability of pressurization refers to the control precision of pressurization during ventilation. It contains three alternatives: (1) maximum pressure drop, the absolute difference between expiratory positive airway pressure (EPAP) and the lowest pressure during inspiration; (2) inspiratory positive airway pressure (IPAP) error, the absolute difference between the actual pressure and the set IPAP during inspiration; (3) EPAP error, the absolute difference between the actual pressure and the set EPAP during expiration.

### Response to Leaks Alteration

Leak correction represents the ability of the ventilator to adapt to the changes of a systematic leak. It has two alternatives: time needed from the moment a leak was increased or decreased until the tidal volume was within 2 standard deviations of the mean tidal volume for each leak level. They were denoted as time to settle (increase) and time to settle (decrease).

### Performance of Reaction

To evaluate the performance of reaction, the following alternatives are considered: (1) Exp T90, the time to accomplish 90% of the drop from peak pressure to EPAP; (2) Insp T90, the time to accomplish 90% of the rise to IPAP; (3) trigger time, point in time at which airway pressure has returned to baseline after downward deflection (start of inspiration effort).

### Volume Delivery

Volume delivery assesses the gas output of a ventilator, includes (1) peak flow rising ratio during inhalation (peak flow divided by Insp T90) and (2) tidal volume.

### Accuracy in Oxygen Delivery

The accuracy in oxygen delivery refers to the difference between the preset oxygen concentration and the actual one delivered.

**Figure 1:**
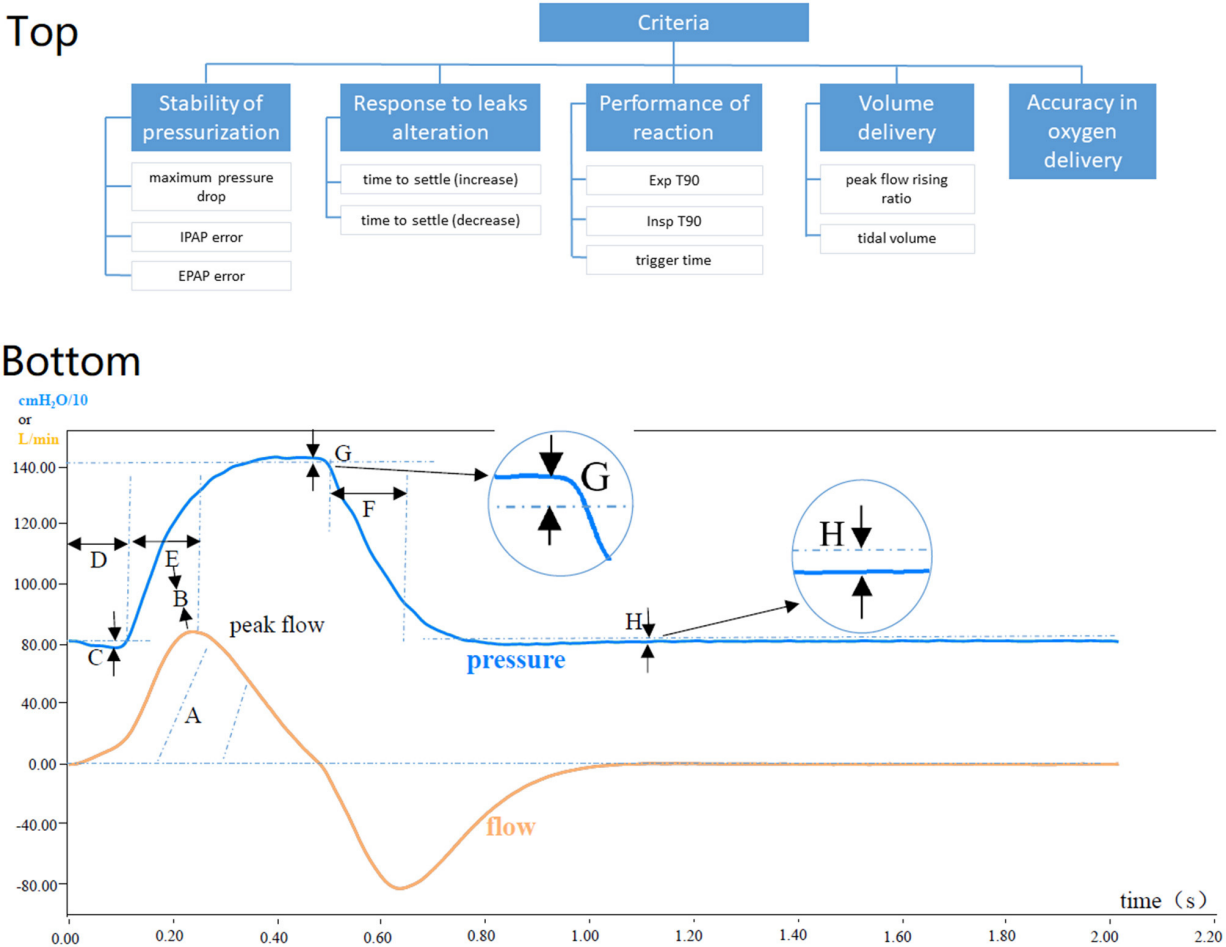
**(Top)** Illustration of the analytic hierarchy process hierarchy. **(Bottom)** explanation of the evaluated parameters. **(Top)** EPAP, expiratory positive airway pressure; IPAP, inspiratory positive airway pressure; Exp T90, the time to accomplish 90% of the drop from peak pressure to EPAP; Insp T90, the time to accomplish 90% of the rise to IPAP. For detailed explanation of the nodes, please refer to the text. **(Bottom)** A, Tidal volume: The volume of gas the user inhales; B, Flow efficiency: Peak flow divided by the length of Insp T90; C, Maximum pressure drop: Deflection of airway pressure from baseline (EPAP) to Pmin; D, Time to trigger: Point in time at which airway pressure has returned to baseline (EPAP) after a downward deflection; E, Insp T90: The time to accomplish 90% of the rise to IPAP; F, Exp T90: The time to accomplish 90% of the drop from peak pressure to EPAP; G, IPAP error: The difference between Ppeak and the set IPAP; H, EPAP error: The difference between the positive end-expiratory pressure and the set EPAP.

The AHP evaluation criteria are illustrated in Figure 1. The relative weights of the nodes in the hierarchy were determined using a survey (https://www.wjx.cn/jq/102986570.aspx.) The participants consisted of senior intensivists (chief physician) and engineers of ventilator manufacturers (>5 years as senior engineer). The participants evaluated the hierarchy through a series of pairwise comparisons that derive numerical scales of measurement for the nodes (i.e., 0 or 1). The criteria are pairwise compared for importance. The alternatives are pairwise compared against each of the criteria for preference. The priorities are derived correspondingly for each node as described in the previous study (11).

Further, eight ICU ventilators with non-invasive ventilation mode and four dedicated non-invasive ventilators were tested according to the scoring system. The features of the tested ventilators are summarized in Table 1. The setting of the ventilators was the same: spontaneous timed mode, 10/min, Inspiratory rise time, when adjustable, was set to the fastest value that did not cause an initial pressure overshoot that would shut down the lung model, and triggering was set at the most sensitive value that did not cause auto-triggering. Four COVID-19 phenotypes were simulated using the ASL5000 lung simulator (IngMar Medical, PA, USA), namely (1) LC, low compliance (compliance of 20 ml/cmH_2_O), (2) IR, increased airway resistance (10 cm H_2_O/L/s), (3) LCIE, low compliance plus increased respiratory effort (respiratory rate of 40 and inspiratory effort of 10 cmH_2_O), and (4) HC, high compliance (compliance of 50 ml/cmH_2_O). All of the ventilators were set to three combinations of IPAP and EPAP levels (10 and 4 cmH_2_O, 20 and 8 cmH_2_O, 30 and 12 cmH_2_O, IPAP and EPAP, respectively). The data were collected at baseline and at three customized leak levels for dedicated non-invasive ventilators (50, 70, and 90 L/min) and for ICU ventilators using non-invasive mode (14, 19 and 25 L/min).

**Table 1:** Summary of ventilators examined in the study.

**Ventilator**	**Connection type (circuit)**	**Manufacturer**	**Leakage compensation (L/min)**	**Trigger type**	**Cycling type**
BiPAP V60	Single	Philips, NL	60	Auto-flow	Auto-flow
Bellavista 1000	Single	Imtmedical, CH	120	Auto-pressure, flow	Auto-pressure, flow, volume
ST-30K	Single	Micomme, CN	120	Auto-flow	Auto-flow
carina	Single	Draeger Medical, DE	50	Manual-pressure, flow	Manual-pressure, flow
VG55	Single	AeonMed, CN	200	Manual-flow	Manual-flow
GA	Single	ResMedandCurative, CN	120	Manual-pressure, flow	Manual-flow
VG70	Double	AeonMed, CN	60	Manual-pressure, flow	Manual-pressure, flow
SV800	Double	Mindray, CN	65	Manual-pressure, flow	Manual-pressure, flow
Servo-I	Double	GetingeGroup, SE	65	Manual-pressure, flow	Manual-flow
R860	Double	GE Medical, US	100	Manual-pressure, flow	Manual-flow
VELA	Double	CareFusion, US	60	Manual-flow	Manual-pressure, flow, volume
V200	Double	Philips, NL	60	Auto-flow	Auto-flow

### Data Collection

**Figure 2:**
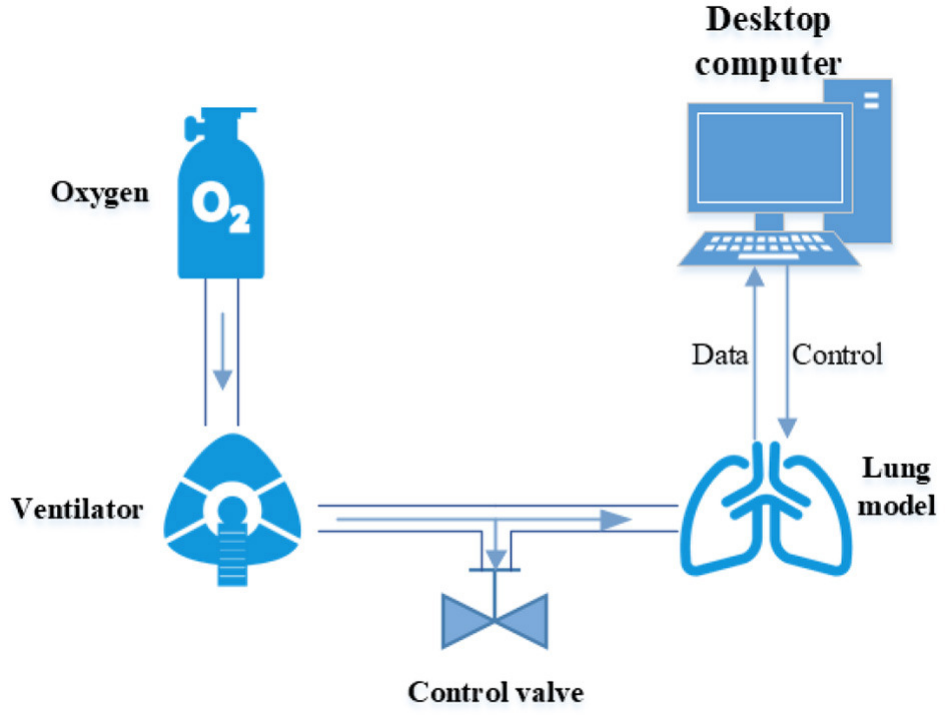
Illustration of test hardware and connection.

Test hardware and its connection are illustrated in Figure 2. The test process consists of four steps: (1) Start the test after setting the lung simulator according to the parameters described previously, and adjustment of the control valve reaching the specified air leakage of the ventilator. (2) Keep the opening of the control valve and restart the lung simulator for testing for 1 min. (3) Repeat the above two steps until three different air leakages have been tested. (4) Dynamic air leakage test was carried out through a control valve with three gears. Data acquisition was done at 512 Hz and stored in a desktop computer.

### Statistical Analysis

Offline analysis on a breath by breath basis was done by the ASL5000 Lab view software (National Instruments, Austin, TX, USA). All breaths were visually inspected and five breaths during equilibrium state were selected for analysis. Outliers were defined as 1.5 times interquartile range. The outliers were eliminated from further analysis. The ranges of the targeted parameters are listed in Table 2. When a ventilator performance was outside of the range, it scored 1 or 0 for better or worse performance, respectively. Mean values of the score were calculated when multiple levels of testing were performed. The overall performance is weight sum of all nodes.

**Table 2:** Ranges of the targeted parameters.

**Parameters**	**score**	**LC**			**IR**			**LCIE**			**HC**		
		**Preset pressure (cmH**_****2****_**O)**											
		**4–10**	**8–20**	**12–30**	**4–10**	**8–20**	**12–30**	**4–10**	**8–20**	**12–30**	**4–10**	**8–20**	**12–30**
Tidal volume (ml)	0	120	200	250	190	350	480	180	184	229	300	400	450
	1	270	400	540	490	780	1,025	416	561	572	600	900	1,200
Peak flow rising ratio (l/s^2^)	0	0			0			0			0		
	1	10			6			15			3		
Trigger time (ms)	0	400			600			400			600		
	1	60			90			60			60
Maximum pressure drop (cmH_2_O)	0	3.55			2.55			7			2.55		
	1	0			0			0			0		
Exp T90 (ms)	0	600			1,000			600			1,400		
	1	100			150			100			100		
Insp T90(ms)	0	900			1,200			900			1,400		
	1	100			150			100			300		
IPAP error (cmH_2_O)	0	±5	±10	±15	±5	±10	±15	±5	±10	±15	±5	±10	±15
	1	0	0	0	0	0	0	0	0	0	0	0	0
EPAP error (cmH_2_O)	0	±2	±4	±6	2	±4	±6	2	±4	±6	2	±4	±6
	1	0	0	0	0	0	0	0	0	0	0	0	0
Time to settle (increase) (s)	0	20											
	1	0											
Time to settle (decrease) (s)	0	12											
	1	0											
Oxygen delivery %	setting	60%											
	0	±10											
	1	0											
	setting	100%											
	0	80											
	1	98											

*IR, increased airway resistance; LC, low compliance; LCIE, low compliance plus increased respiratory effort; HC, high compliance*.

## Results

The survey was sent out to 66 clinical and engineering experts. The weighting factors were defined based on 54 valid replies (16 from senior intensivist and 38 from engineers). The final weights of the nodes are summarized in Table 3. The nodes of the criterion “pressurization” had similar weights. The experts considered time to settle (increase) and tidal volume much more important than the time to settle (decrease) and peak flow rising ratio, respectively. Trigger time was the most important node in the criterion “reaction”.

**Table 3:** The final weights of nodes of the scoring system.

**First level criteria**		**Second level criteria**	
The stability of pressurization	0.223	Maximum pressure drop	0.337
		EPAP error	0.278
		IPAP error	0.385
Response to leaks alteration	0.229	Time to settle (increase)	0.622
		Time to settle (decrease)	0.378
Volume delivery	0.160	Peak flow rising ratio during inhalation	0.385
		Tidal volume	0.615
Performance of reaction	0.242	Insp T90	0.341
		Exp T90	0.160
		Trigger time	0.498
Accuracy in oxygen delivery	0.146	–	–

**Figure 3:**
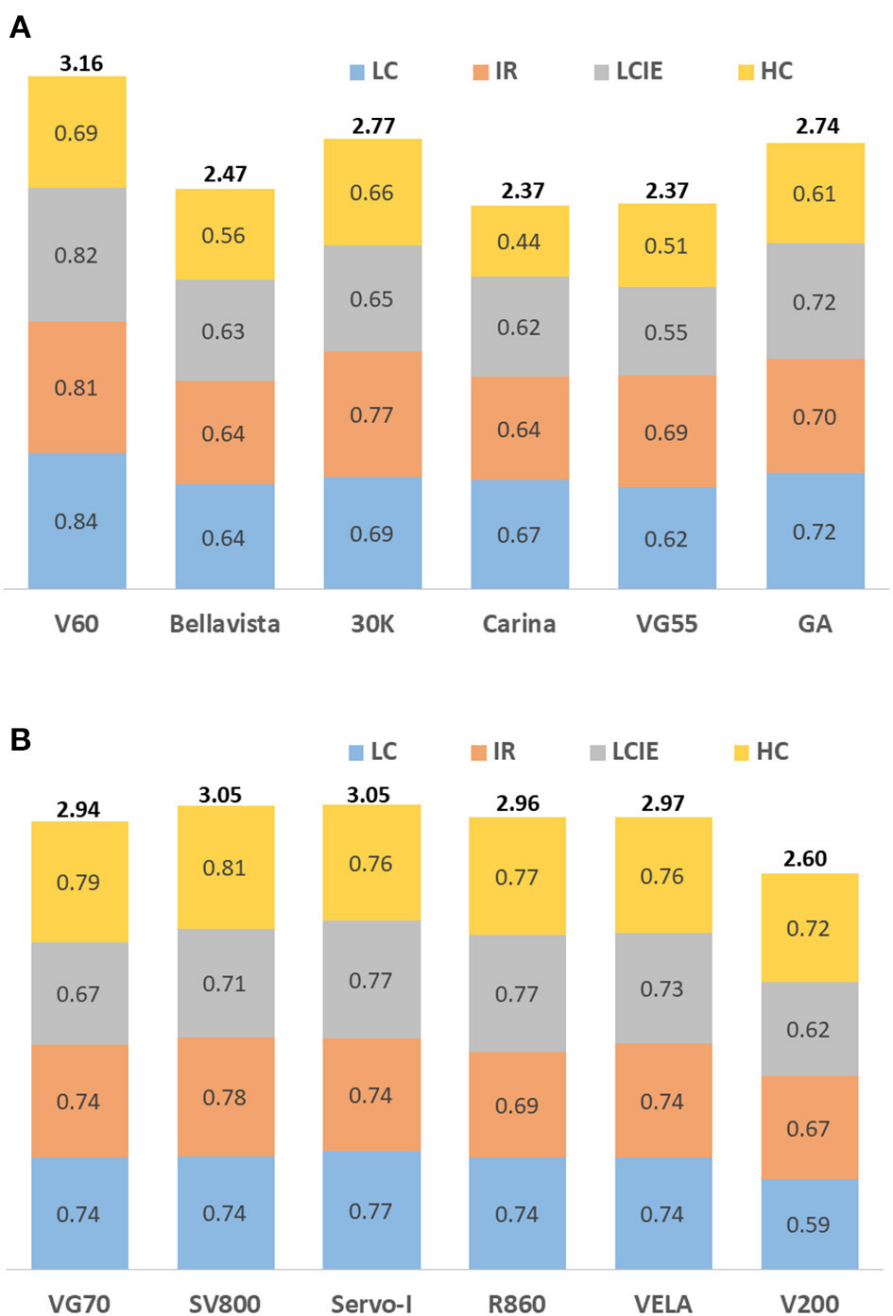
Overall performances of the ventilators under three simulated COVID-19 phenotypes. IR, increased airway resistance; LC, low compliance; LCIE, low compliance plus increased respiratory effort; HC, high compliance. **(A)** non-invasive ventilators with single-circuit connection. **(B)** ICU ventilators with non-invasive ventilation mode and double-circuit connection.

**Figure 4:**
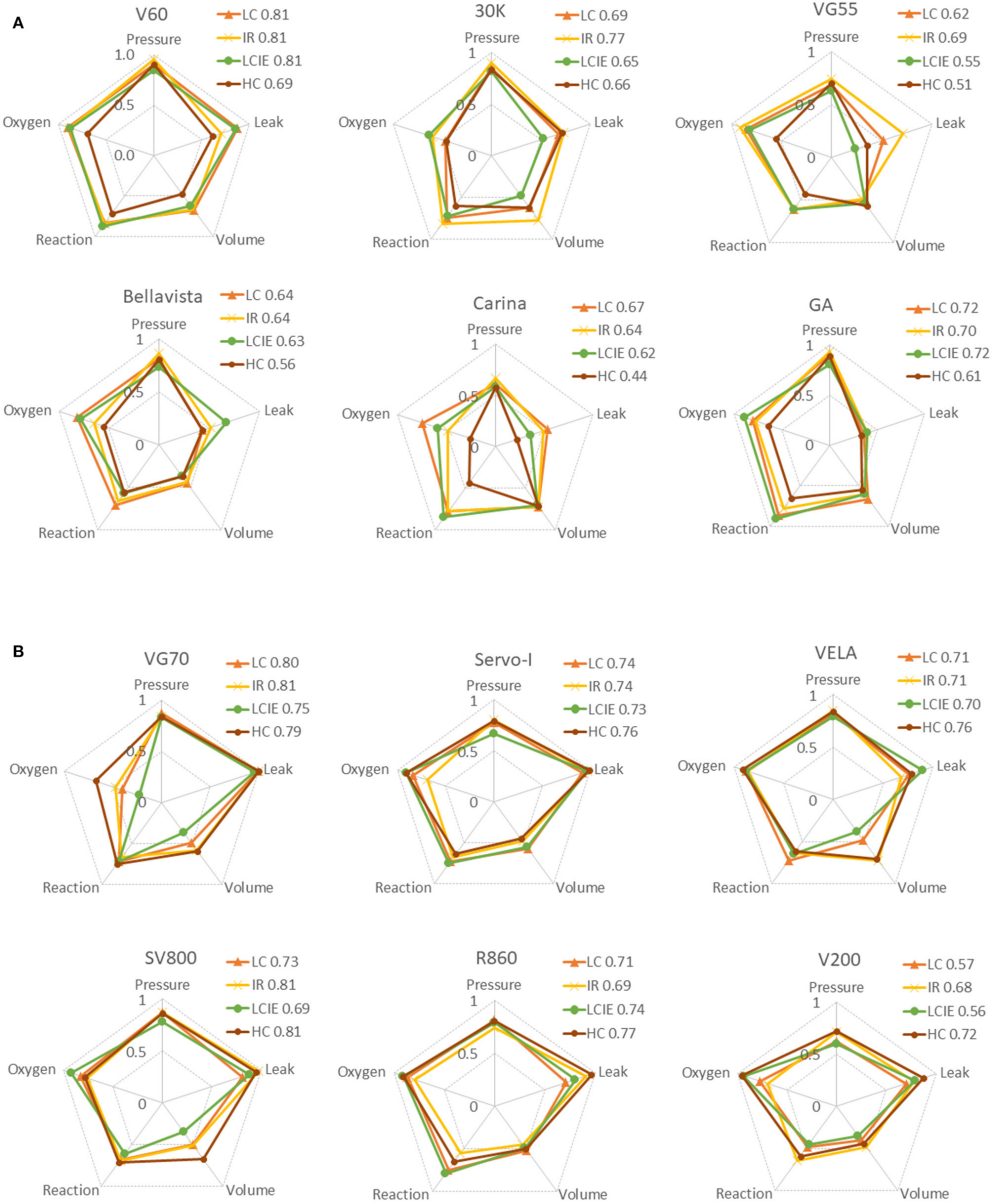
Radar charts summarizing the main criteria of the performance evaluation. Data are expressed as the score calculated based on the proposed scoring system. Scores for three simulated COVID-19 phenotypes are superimposed on the same chart (IR, increased airway resistance; LC, low compliance; LCIE, low compliance plus increased respiratory effort; HC, high compliance). The following five characteristics are summarized for each ventilator (clock-wise): the stability of pressurization (pressure), response to leaks alteration (leak), volume delivery (volume), performance of reaction (reaction), and accuracy in oxygen delivery (oxygen). **(A)** Non-invasive ventilators with single-circuit connection. **(B)** ICU ventilators with non-invasive ventilation mode and double-circuit connection.

Overall performances of the ventilators under four simulated COVID-19 phenotypes were summarized in Figure 3. V60 and Servo-I have the best overall performance among the ventilators tested (for single and double circuits). Some ventilators have stable performance in different simulated phenotypes whereas the others have over 10% scoring differences (e.g., 30 K and V200). Nevertheless, the overall performances among the ventilators for double circuits were similar. The performances of the ventilators in five criteria were illustrated using radar charts (Figure 4). Significant inaccuracies and variations in performance among different ventilators were observed, especially in the aspect of response to leaks alteration, oxygen, and volume delivery. For example, Servo-I had excellent performance in response to leaks alteration in all simulated phenotypes, however, its performance in volume delivery was much weaker. The accuracy in oxygen delivery for VG70 strongly depended on the simulated phenotypes.

## Discussion

In the present study, we demonstrated the process of establishing a scoring system to evaluate the overall performance of ventilators. Further, with help of the proposed scoring system, 12 ventilators were evaluated. It was found that the performance of ventilators depended on targeted lung models and varied significantly among different investigated criteria.

**Figure 5:**
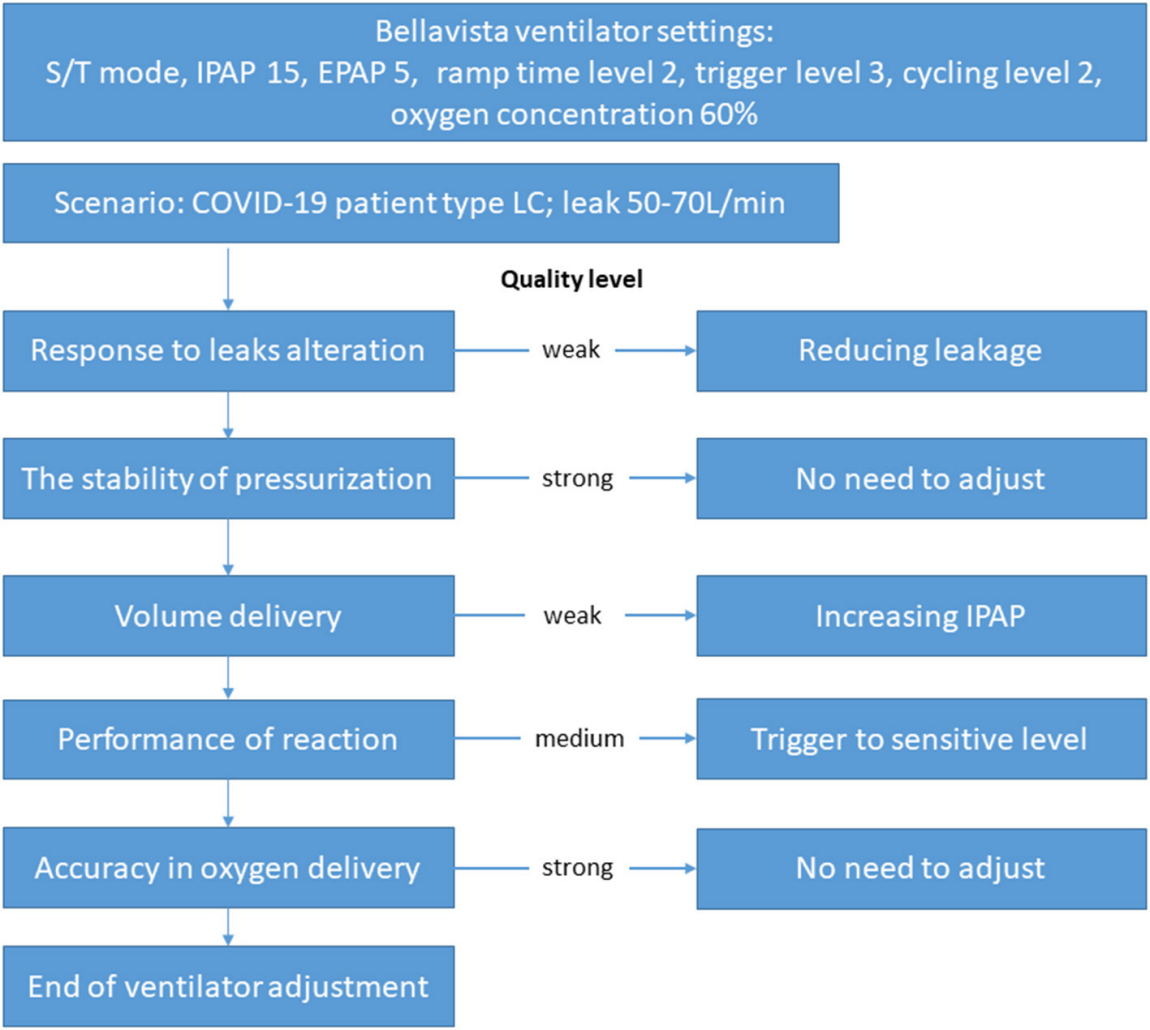
Example on how the findings of the current study could be used in clinical practice. The ventilator Bellavista was used in a patient with lung status similar to the low compliance (LC) model we simulated in the current study. Leakage measured was ~50–70 L/min under the presented ventilator settings. Based on the quality levels we found, the ventilator settings should be adjusted accordingly.

COVID-19 may affect the respiratory system in various ways. In patients with respiratory failure, the oxygen level may drop to a low level that meets the definition of acute respiratory distress syndrome. However, the respiratory system compliance may still be normal (7). The compliance may decrease during disease progression, which might be caused by inappropriate settings of ventilator (12). If the performance of the ventilator is unacceptable with large discrepancy between set values and actual ones, ventilator-induced lung injury might occur even the ventilator settings were optimized. In combination with other existing lung diseases in the patients, such as chronic obstructive lung disease, more frequent monitoring and modified respiratory therapy are required in ICU (13). In the present study, we simulated four phenotypes of COVID-19, with possibly increase of airway resistance (IR), decrease of compliance (LC), increase of inspiratory effort (LCIE), or HC. The ventilators evaluated in the study showed different performances under different lung models. In clinical practice, if more than one type of ventilator was available, the one that best fits the underlying disease should be selected. If only limited types of ventilators are at disposal, intensivist, or respiratory therapist should be aware of the pitfalls of the ventilators during application. Taking the ventilator Bellavista for example, when it is applied on COVID-19 patient with LC, and the measured leakage is ~60 L/min, we suggest that the ventilator should be adjusted according to the findings of the present study (Figure 5). In particular, response to leaks alteration and volume delivery of this ventilator scored low. Leakage reduction and IPAP increase should be considered in this scenario.

The current pandemic has created medical resource scarcities, especially in regions where ventilators and trained personnel are already in short supply. Many new attempts of ventilator manufacturing were presented, including some low cost ventilator (14), and shared ventilator setup for multi-patient simultaneous use (15). The scoring system established in the present study should be able to help evaluating the performance of ventilators in a standard manner. Next generation of ventilator is toward physiological closed-loop systems (16). Decision making would be still in the hands of physicians but with the extensive physiological monitoring in current clinical environment, a physiological parameter could be accurately fed back to the controller and solve the high-stress environments as COVID-19 pandemic with a shrinking workforce (17). To develop the correct feedback loop, a full understanding of the ventilator performance is required. The current study might be a step toward the physiological closed-loop system. Besides, the proposed scoring system and the models simulated by ASL5000 may help the medical students to further understand the interaction between patients and ventilator in addition to mannequin-based and computer screen-based simulation (18).

The following limitations are acknowledged. (1) The five criteria of the AHP hierarchy (and the corresponding alternatives) were predefined. Only these aspects of ventilator performance were evaluated and considered in the scoring system. Some importance aspects could have been missed when we designed this scoring system for non-invasive ventilation. For invasive ventilation mode, other parameters should be considered as well. Nevertheless, the knowledge and procedure of building the scoring system can be easily transferred. (2) The definition of the parameter value ranges in Table 2 might have influence on the overall score of particular ventilator. But if the same ranges are used for all comparison, the scores of the ventilators are comparable. (3) This was a lung model study with limited number of variations simulating COVID-19 patients. We demonstrated the performance of various ventilators under the preselected scenarios. The study design was not intended to validate the scoring system. In the future study, actual outcomes and influences on real subjects could be considered.

Clinicians should be aware of possible strength and weakness of ventilators. Performance of other ventilators can be conducted using the scoring system developed in the present study.

It is feasible to use the proposed scoring system to evaluate the ventilator performance. In the COVID-19 pandemic, clinicians should be aware of possible strength and weakness of ventilators.

## Data Availability Statement

The original contributions generated for the study are included in the article/supplementary material, further inquiries can be directed to the corresponding author/s.

## Author Contributions

XH had full access to all of the data in the study and takes responsibility for the integrity of the data and the accuracy of the data analysis. XH, FX, KW, and HG had designed the study and analyzed the data, and revised the manuscript significantly. GM, RW, YZ, and QY had collected the data and revised the manuscript significantly. ZZ and LX contributed to study design and data interpretation, and drafted the manuscript. All authors contributed to the article and approved the submitted version.

## Conflict of Interest

The authors declare that the research was conducted in the absence of any commercial or financial relationships that could be construed as a potential conflict of interest.

## Abbreviations

ICU: intensive care units
COVID-19: the novel coronavirus disease 2019
AHP: analytic hierarchy process
EPAP: expiratory positive airway pressure
IPAP: inspiratory positive airway pressure.
